# Cancer Stem Cells Connecting to Immunotherapy: Key Insights, Challenges, and Potential Treatment Opportunities

**DOI:** 10.3390/cancers17132100

**Published:** 2025-06-23

**Authors:** Sivapar V. Mathan, Rana P. Singh

**Affiliations:** 1 Cancer Biology Laboratory, School of Life Sciences, Jawaharlal Nehru University, New Delhi 110067, India; sivaparmathan@gmail.com; 2 Department of Biotechnology, Gautam Buddha University, Greater Noida 201312, India; 3 Department of Pharmaceutical Sciences, Skaggs School of Pharmacy and Pharmaceutical Sciences, University of Colorado, Anschutz Medical Campus, Aurora, CO 80045, USA

**Keywords:** cancer stem cells (CSCs), CSC niche, tumor microenvironment (TME), immunotherapy, immune evasion

## Abstract

Cancer continues to pose a significant challenge to global health, resulting in millions of deaths annually despite advancements in treatments like surgery, chemotherapy, and radiotherapy. A key factor complicating successful outcomes is the presence of cancer stem cells (CSCs), which possess distinctive features that facilitate tumor initiation and progression as well as resistance to therapies. These cells are adept at evading conventional treatments and can hinder the effectiveness of immunotherapy, often manipulating the tumor microenvironment to suppress immune responses. This review delves into the complex interplay between CSCs and immune cells, emphasizing their contributions to tumor heterogeneity and therapeutic resistance. By investigating the CSC niche in which these cells thrive and their complex interactions with the immune system, we aim to reveal new therapeutic avenues that could enhance patient outcomes and minimize the risk of recurrence. CSCs are characterized by remarkable self-renewal and plasticity, allowing them to transition between stem-like and differentiated states in response to various stimuli. Their existence within the CSC niche confers immune protection and maintains stem-like properties while promoting immune evasion. Activating key signaling pathways and specific surface markers is crucial in developing CSC traits, pointing to potential strategies for effective tumor eradication. Conventional therapies often fail to eliminate CSCs, which can lead to tumor recurrence. Therefore, innovative immunotherapeutic strategies such as dendritic cell vaccines (DC vaccines), chimeric antigen receptor (CAR) engineered T cells, and immune checkpoint inhibitors (ICIs) are under examination. This review sheds light on CSC’s roles across different malignancies, highlighting the necessity for innovative targeted approaches in cancer treatment.

## Simple Summary

Cancer presents a serious global health challenge, with millions of lives lost each year despite advances in treatment methods such as surgery, chemotherapy, and radiotherapy. One significant obstacle in overcoming cancer is the presence of cancer stem cells (CSCs). These unique cells enable tumors to grow and spread while also resisting conventional therapies, often undermining the effectiveness of immunotherapy. CSCs can manipulate their environment to evade immune responses, complicating treatment efforts. This review highlights the relationship between CSCs and the immune system, exploring how they contribute to tumor diversity and treatment resistance. CSCs are known for their remarkable ability to self-renew and adapt, switching between stem-like and more differentiated states in response to various factors. This adaptability helps them survive traditional therapies that target rapidly dividing cells. Moreover, new immunotherapeutic strategies are being explored to tackle CSCs, including dendritic cell vaccines, CAR T-cell therapies, and immune checkpoint inhibitors. These approaches aim to eliminate CSCs more effectively and reduce the chances of cancer relapse. This review emphasizes the ongoing research into CSCs across different cancers. It underscores the need for innovative treatment strategies specifically targeting these resilient cells, ultimately improving patient outcomes and minimizing recurrence risks. Addressing the challenges posed by CSCs is crucial for the future of effective cancer treatment.

## 1. Introduction

**Figure 1:**
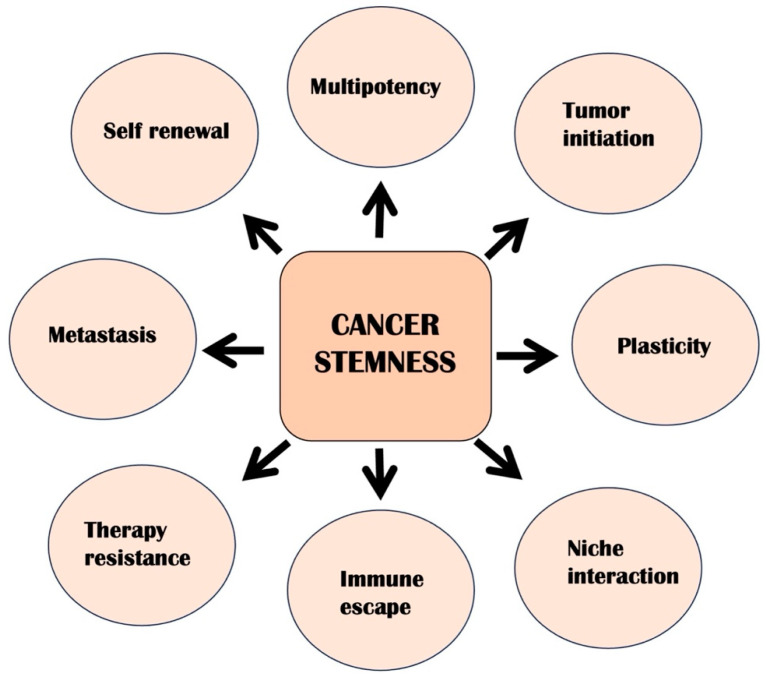
Characterizing cancer stem cells: fundamental hallmarks emerging attributes and related features.

Cancer represents a significant global health challenge and ranks as one of the primary causes of mortality worldwide. In the United States (US), it stands out as the second leading cause of death. Among various cancer types, lung cancer holds the unfortunate distinction of being the most frequently occurring form of cancer in men. It is the leading factor contributing to male cancer-related deaths. Breast cancer ranks as the most frequently diagnosed malignancy and is the primary cause of cancer-related mortality within this group. While breast cancer poses a significant health threat to women, it is essential to recognize that on a global scale, lung cancer accounts for the greatest number of fatalities, followed by colorectal, liver, breast, and gastric cancers. In the US, cancer incidence rates have decreased in men, and they have increased in women. Due to significant advances in treatment, early detection, and smoking cessation, the cancer mortality rate has declined [1,2]. Over the past few decades, cancer treatment and management have improved significantly. Standard treatment approaches for cancer typically include surgical intervention, chemotherapy, and radiation therapy. These methods form the cornerstone of cancer management, each playing a crucial role in targeting and eliminating cancer cells. These traditional therapies are quite effective in the treatment of cancer. Still, challenges such as metastasis, tumor relapse or recurrence, and acquiring resistance during treatment decrease the overall success rate of cancer therapy. In cancer biology, cancer stem cells (CSCs) are essential players in various aspects of cancer, including the initiation and advancement of tumors. They contribute to the spread of cancer cells to other body parts, are involved in immune response evasion, and are often responsible for relapses after treatment. Additionally, CSCs are a significant factor in developing resistance to therapies, making them a critical focus for research and treatment strategies (Figure 1) [3]. CSCs can self-renew and are also known as tumor-initiating cells, as they have high tumorigenic potential and can seed tumor initiation. CSCs are dormant and can sustain the growth of tumor cells. Conventional therapies generally target rapidly dividing malignant cells, and since CSCs are slow-dividing and remain quiescent, they evade conventional therapies.

Cancer stem cells are characterized by several key traits that set them apart. Notably, they possess a unique ability to initiate tumor growth when injected into immunocompromised mice and a remarkable capacity for self-renewal. Additionally, CSCs can differentiate into various cell types, contributing to tumor complexity. These defining characteristics are essential in understanding CSCs’ role in cancer biology. Traditionally, CSCs are thought to be part of well-defined hierarchical populations. However, recent findings indicate that even differentiated cancer cells can acquire stem-like properties under certain conditions. This observation highlights the concept of plasticity as an emerging hallmark of cancer stemness, suggesting that these cells can adapt and evolve in response to their environment. The distinctive features of CSCs not only play a crucial role in their ability to resist therapeutic interventions but also contribute to metastasis. Consequently, therapy resistance and the potential for cancer spread are recognized as enabling attributes that arise from these hallmark characteristics. Moreover, the interactions between CSCs and their microenvironment and their ability to evade immune detection are identified as associate features. While these aspects are significant to understanding CSC behavior, they are not directly categorized within the primary hallmarks of cancer stemness. The interplay of these elements underscores the complexity of CSCs in the context of cancer progression and therapeutic resistance.

CSCs are dynamic and highly adaptable to treatment pressures. These cells can transition between a stem-like state and a differentiated state depending on metabolic or therapeutic stress, hypoxia, or other external stimuli in the tumor microenvironment (TME). This phenomenon is known as CSC plasticity. In the TME, under certain conditions, non-CSCs dedifferentiate into cancer stem-like cells, which leads to therapeutic resistance. CSCs resist chemotherapy by activating drug efflux mechanisms, overexpressing anti-apoptotic genes, engaging DNA damage repair mechanisms, and remaining quiescent. CSCs contribute to tumor heterogeneity and evade the tumor immune response. Gaining insights into the plasticity of CSCs and how they influence tumor advancement and resistance to treatment is essential for creating new and effective therapeutic strategies [4].

Immunotherapy represents a groundbreaking cancer treatment strategy that leverages our immune system to target and eliminate cancerous cells. By activating immune responses or augmenting the immune system’s capacity to identify and destroy tumors, this approach holds significant potential for enhancing patient outcomes in cancer care. Immunotherapy includes adoptive cell therapies (ACTs) and immune checkpoint inhibitors (ICBs), significantly changing the cancer treatment landscape. However, developing intrinsic and acquired resistance to these approaches has created challenges, highlighting the incomplete understanding of how tumors evolve during these immunotherapeutic strategies. CSCs contribute notably to the challenge of therapy resistance. They can evade the immune system’s detection by releasing immunosuppressive cytokines. This secretion not only attracts immune regulatory cells, such as myeloid-derived suppressor cells (MDSCs) and regulatory T cells (Tregs), but also diminishes the major histocompatibility complex (MHC) molecules’ expression, further hindering the body’s ability to mount an effective immune response against the tumor. CSCs can proliferate and differentiate into different types of cells and evade immunotherapy by interacting with stromal cells and other immune cells in the tumor microenvironment, which leads to tumor survival and immunosuppression. CSCs exist in a unique microenvironment, the CSC niche, comprising fibroblasts, stromal cells, pericytes, endothelial cells, metabolites, cytokines, extracellular matrix (ECM), etc., protecting CSCs from therapeutic drugs and immune response [5]. This review shows the critical relationship between CSCs and immunotherapy and highlights the present challenges, recent developments, and potential therapeutic avenues. By specifically targeting the CSCs and understanding their resistance mechanisms, we can improve the therapeutic efficacy, decrease tumor recurrence, and improve overall patient outcomes.

## 2. Cancer Stem Cells

**Figure 2:**
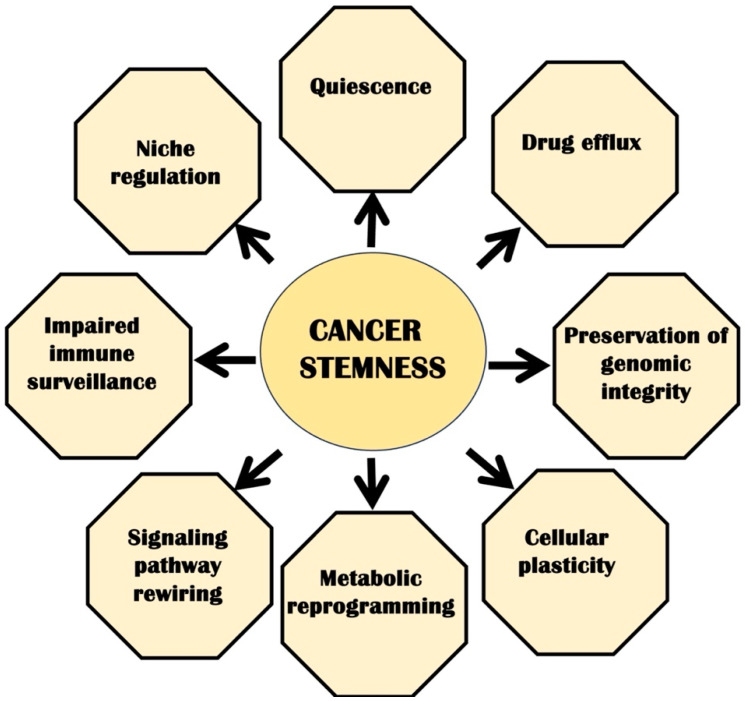
Cancer stem cell properties in therapeutic resistance.

The concept of stem-like cells in tumors emerged during the 1960s–70s [6,7]. Later, the idea of CSCs originated in the 1990s in acute myeloid leukemia (AML), identifying a unique group of undifferentiated cells with self-renewal ability and the ability to initiate tumor formation [8]. This groundbreaking discovery of stem-like cells sparked exploration into the presence of similar stem-like cells across different cancers. CSCs are present in all solid tumors, including breast [9], brain [10], pancreas [11], colon [12], melanoma [13], liver [14], and gastric cancers [15,16]. Several studies have shown that the presence of CSCs correlates with poor survival rates of patients with cancer [17]. CSCs share similar characteristics with normal stem cells, and they acquire regenerative potential through abnormal signaling pathways, which vary among cancer types. For example, CSCs in breast cancer express the CD44s variant, which activates PDGFRβ/STAT3 signaling [18]. In glioblastoma, it depends on distinct signaling pathways to sustain their growth, which shows complex regulation of CSCs in different cancers [19]. CSCs are distinguished by several key features, including their exceptional capacity for self-renewal, potential to form tumors, and the ability to switch between a stem-like phenotype and a more differentiated state. CSC plasticity increases their survival against therapies and makes them adapt to the altering TME during cancer progression. Recently, studies have identified additional other complex regulatory mechanisms in CSC regulation, including like RNA editing, splicing, methylation, and chromatin remodeling, in leukemia [20], chronic myeloid leukemia (CML) [21], glioblastoma [22], and AML [23]. Studies have reported that CSCs can migrate from the primary tumor and form secondary tumors in distant locations. The occurrence of cancer stem cells (CSCs) at the invasive margins of tumors is linked to decreased survival rates among patients. These CSCs play a significant role in altering drug metabolism and promoting the epithelial-mesenchymal transition (EMT), which can result in resistance to treatment. They are characterized by a high expression of ATP-binding cassette (ABC) transporters, proteins that aid in expelling drugs from the cells, ultimately contributing to multidrug resistance (MDR) [24]. Furthermore, the adaptive nature of CSCs enables them to transdifferentiate into various cell types, enhancing their ability to survive under therapeutic pressure (Figure 2). CSCs transdifferentiate into endothelial cells and pericytes, which have been reported in breast cancer [25], renal carcinoma [26], and glioblastoma [27,28].

CSCs are resistant to various forms of therapies. CSCs utilize multiple strategies to evade traditional treatments, which include entering a dormant or quiescent state, increasing the efflux of therapeutic agents, and preserving the integrity of their genetic material. This adaptability allows CSCs to modify their metabolic and signaling pathways, thereby overcoming the inhibitory effects of targeted therapies. CSCs have enhanced abilities to evade the immune system, making them less responsive to immunotherapy. When faced with therapeutic challenges, CSCs can maintain and restore their undifferentiated state in the CSC niche.

## 3. Cancer Stem Cells and Their Role in Immunotherapy Resistance

The dynamics in the tumor microenvironment involve the complex interplay between innate immune cells like natural killer (NK) cells, MDSCs, dendritic cells (DCs), and adaptive T and B cells. The unique characteristics of CSCs make them either susceptible or resistant to immune cells. CSCs express low levels of MHC-I molecules, which makes them susceptible to NK cells [29]. To create an immunosuppressive TME, CSCs secrete various cytokines that alter immune cells. CSCs secreted cytokines polarize M1 tumor-associated macrophages (TAMs) to the M2 phenotype. M1 macrophages are known to have anticancer properties, and M2 macrophages are known to be immunosuppressive and promote tumor growth [30]. CSCs induce the secretion of interleukin-10, differentiating Treg cells, which are immunosuppressive, thus promoting tumor progression [31]. Understanding the interaction between immune cells and CSCs in TME is crucial since this enables them to survive, suppress, and evade the tumor immune response. This poses a significant challenge to cancer treatment as it contributes to therapeutic resistance and tumor recurrence.

### 3.1. Interactions Between CSCs and Immune Cells

Dendritic cells function as primary APCs that display TAAs on MHC-I molecules, initiating immune responses. However, CSCs can disrupt the maturation of DCs or increase the tolerogenic DCs by secreting TGF-β1 [32]. This interaction reduces MHC-II expression and inhibits the production of costimulatory molecules, such as CD80 and CD86 [33,34,35]. Furthermore, CSCs can release extracellular vesicles (EVs) that carry MHC-I and HLA-G, which hinder the maturation of DCs through the STAT3 signaling pathway [36,37]. Regulatory DCs interact with CSCs via CXCL-12 binding to CXCR-4, which helps maintain the self-renewal of CSCs [38]. Additionally, CXCL-1^+^ DCs regs can promote stemness signaling in colon cancer cells, enhancing their metastatic potential [39]. Interactions between CSCs and tumor-associated macrophages foster an immunosuppressive TME [40]. The TME surrounding CSCs is rich in cytokines, extracellular matrix components, TGF-β, and periostin, facilitating macrophage recruitment and polarization [41]. Periostin expressed on CSC membranes helps attract monocytes from the bloodstream and converts them into TAMs, supporting the survival of CSCs in the TME [42]. TGF-β1 generates EpCAM^+^ CSCs, which promote invasion and metastasis in HCC by triggering the EMT [43]. Moreover, TAMs can induce increased CD47 expression on various CSCs, such as leukemia, HCC, and pancreatic cancer [44,45,46]. CD47 binds SIRPα on macrophages, which helps protect CSCs from being eliminated by the immune system through phagocytosis. Additionally, signals from TAMs can stimulate the expression of immune checkpoints [47]. Thus, the interplay between CSCs and TAMs establishes an immunosuppressive TME that assists CSC survival and complicates tumor eradication through immunotherapy.

MDSCs release various chemokines and cytokines that can dampen the efficacy of immunotherapeutic approaches. In CSCs, mTOR signaling enhances the infiltration of MDSCs [48]. CSCs can trigger the expression of TGF-β, leading to the recruitment of MDSCs at tumor sites in melanoma [49]. Furthermore, in LSCs, TIM-3 and galectin-9 can increase the presence of TAMs and MDSCs, weakening anti-tumor immune responses [50]. MDSCs secrete exosomal S100A9, enhancing STAT3 and NF-κB signaling, and they promote cancer stemness and survival through the upregulation of piRNA-823 [51,52]. These interactions between CSCs and MDSCs can contribute to enhancing cancer stemness and promoting tumor growth and progression. Interactions between CSCs and Treg cells foster the formation of the TME. In glioblastoma, CSCs express TGF-β1 and PD-L1, facilitating the infiltration of Treg cells. CSCs recruit Tregs via CCL1 secretion, which in turn produces TGF-β1 and IL-17, enhancing the self-renewable ability of CSCs [53,54,55]. Gastric CSCs support the development of CSCs via STAT3 signaling while simultaneously evading T-cell recognition [56]. Tregs also produce VEGF to sustain the self-renewal, cancer stemness, and survival of CSCs in a hypoxic environment [57]. Moreover, Tregs release cyclooxygenase-2, which impairs effector T-cell function in PGE-2-dependent manner, further illustrating how CSC–Treg cell interactions promote immune evasion and contribute to the challenges faced in cancer immunotherapy [58].

Typically, T cells identify TAAs on presented APCs as MHC–peptide complexes. However, CSCs can decrease the expression of TAAs and MHC-I, induce different MHC-I allelic variants, and increase the expression of PD-L1 to evade immune detection [31,59,60,61,62]. Decreased MHC-I expression impacts T-cell activation [63]. CSCs have been shown to upregulate key factors such as VEGF, PD-L1, and TIM-3 in hypoxic conditions [64]. During the progression and metastasis of human neural crest cells, CSCs can be found at tumor invasion sites, where they evade the antitumor immune response by obstructing CD8^+^ T-cell infiltration [65,66]. Similarly, prostate CSCs can inhibit T-cell activation and cytokine production through the expression of galectin-3, thereby shielding themselves from destruction by cytotoxic T cells [67]. Additionally, quiescent CSCs can diminish T cells’ ability to recognize and eliminate tumor cells by decreasing the expression of NLRC5, a transactivator involved in the MHC-I-mediated immune response [68]. On the other hand, NK cells possess a receptor known as NKG2D, which facilitates the targeting and destruction of CSCs that lack MHC-I through a mechanism that does not rely on APCs [69]. For instance, NKG2D-expressing NK cells can eliminate MHC-I-negative colon CSCs, while those expressing NKp30 and NKp44 can directly attack and eliminate MHC-I-negative ovarian CSCs [29,70]. Moreover, CSCs increase the expression of HLA-G, which then interacts with inhibitory ligands on NK cells, such as KIR2DL4 and NKG2A. This interaction renders CSCs less susceptible to NK-mediated lysis by inhibiting NK cell activation [71,72,73]. Furthermore, CSCs that express SOX2 or SOX9 have been found to downregulate the expression of NKG2D ligands, thereby escaping immune clearance by NK cells [74]. As a result, CSCs can develop resistance to NK cell-targeted therapies by increasing MHC-I, contributing to tumor recurrence [75]. In conclusion, a deeper understanding of the mechanisms behind NK cell-mediated removal of CSCs may offer valuable insights for developing targeted immunotherapies to eradicate these resilient cancer cells.

### 3.2. Immune-Mediated Tumor Dormancy and Intratumor Heterogeneity

Understanding tumor biology requires a deep dive into immune-mediated dormancy, where the immune system keeps cancer cells in check, preventing their progression without completely eradicating them. Studies have shown that adaptive immunity can effectively maintain this dormancy allowing the hidden cancers to remain subdued over prolonged periods [76]. This phenomenon forms part of the broader narrative of cancer immunoediting. The immune system not only suppresses tumor growth but also shapes the immunogenic characteristics of tumors over time. The immune system has a dual role in cancer progression, a well-documented phenomenon that supports the concept of immunoediting [77]. Immunoediting consists of three primary phases. In elimination, the immune system often eradicates nascent tumors via innate and adaptive responses before they become detectable. In equilibrium, tumors are kept in a state of functional dormancy, maintained by a balance between antitumor or tumor-promoting factors. In escape, tumor cells proliferate unchecked when the immune response fails to control their growth, culminating in an immunosuppressive environment that allows for clinically detectable diseases [62,78].

Immune-mediated dormancy closely resembles the equilibrium phase, characterized by the same balance between immune antitumor actions and protumor influences. A poignant example involved kidney transplant recipients who developed melanoma [79]. This case demonstrates the immune system’s remarkable ability to sustain tumors in a dormant state within the non-primary organs. In preclinical studies utilizing the sarcoma mouse model, the maintenance of tumor dormancy was shown to involve several adaptive immune components, including T cells, IL-12, and IFN-γ [76]. Additional factors, such as Tregs, MDSCs, NK cells, and cytokines significantly impact the control of dormant disseminated tumor cells. Moreover, it is critical to recognize the non-immune mechanisms, which may also promote tumor cell dormancy [80]. These insights indicate that transforming cancer into a chronic, dormant state may be a more attainable therapeutic objective than completely eradicating CSCs. Differentiated tumor cells, which comprise the majority of the tumor mass, tend to be more susceptible to immune responses. Consequently, immunotherapy could be more effective in targeting these cells while allowing CSCs to remain in a dormant state. Their resilience and adaptability hinder the complete elimination of cancer cells. However, gaining control over both actively dividing and dormant cancer cells holds promise in transforming cancer into a manageable and chronic condition.

Intratumor heterogeneity (ITH) adds another layer of complexity to the treatment landscape. Two models have been proposed to explain the heterogeneity within the tumor. One is the clonal evolution model, which posits that random mutations occurring in the individual tumor cells allow for the adaptation and selection of most fit clones. According to this model, ITH arises through natural selection. Clones that acquire advantages in growth are likely to proliferate, while those with reduced fitness may be out-competed and potentially face extinction. Significantly, these clonal advantages may vary in different regions and at different times, as distinct environmental pressures in specific regions of tumor can lead to the emergence of specific hypoxia-adapted clones in oxygen-deprived regions or fast-growing clones in nutrient-rich regions. Throughout the progression of the disease, these clones can undergo spatial and temporal changes, contributing to the sub-clonal architecture, which is further complicated by therapeutic interventions [81,82,83].

In the CSC model, only a subset of cancer cells can self-renew indefinitely, thereby driving tumor growth. Tumors are organized in a hierarchical structure akin to normal tissues, with a foundation based on healthy stem cells. In this hierarchy, CSCs generate cellular diversity through a differentiation process, creating various cell types within the tumor [84]. This hierarchical structure is not linear; it can exhibit plasticity, allowing differentiated cells to revert to a stem-like state under certain conditions [85,86]. This concept of cellular plasticity helps integrate the clonal evolution and CSC models, as mutations in differentiated cells can grant them self-renewable capabilities, thus establishing hierarchical CSC clones and enhancing the functional diversity present within the tumor [87,88]. Evidence suggests that CSCs likely originate from tissue-resident stem cells, as transforming normal cells to malignant ones requires multiple mutations, which are more feasible in long-lived stem cells [89]. Studies suggest that cancer risk is correlated with the number of divisions of stem cells, implying that they may be the source of many tumors [90]. Recognizing tissue-resident stem cells as potential precursors to CSCs supports the CSC hypothesis and highlights the importance of investigating the immunological characteristics of CSCs versus those of normal stem cells. Both possess immune-evasive properties likely inherited from their origins, where immune evasion is vital for tissue maintenance. Studies indicate that quiescent stem cells downregulate antigen presentation, enabling them to evade immune detection a trait that may persist in CSCs [68].

Identifying CSC-like cells poses significant challenges due to tumor heterogeneity, a lack of universal markers, and methodological inconsistencies [91,92]. While the CSC model advanced cancer research, its application and validation are still being explored. Studies have shown that specific cell populations exhibit stem-like properties, including self-renewal and tumor initiation, but these findings are often context-dependent. For example, in solid tumors like melanoma, the frequency of tumor-initiating cells can vary significantly, complicating the concept of a definitive CSC population [93]. ITH refers to the genetic variation present within a single tumor, encompassing both clonal and subclonal mutations. This diversity plays a crucial role in how cancer develops and responds to treatments [94]. Exposure to UVB light leads to increased tumor heterogeneity, which in turn negatively impacts the immune response in melanoma. Tumors exhibiting high ITH tend to show a decrease in immune cell infiltration and a weakening of antitumor immunity. This reduced immune effectiveness can be traced back to the presence of subclonal mutations, which the immune system may not effectively recognize due to their limited expression within the tumor population [95]. Studies have shown that tumors characterized by high levels of ITH are frequently associated with subclonal mutations that can evade detection, contributing to therapy resistance [96]. This highlights the necessity of incorporating ITH considerations into the development of immunotherapeutic approaches [97]. CSCs are known to play a pivotal role in ITH through their ability to generate diverse populations [98]. Alterations found in CSCs are expected to be present across all cancer cells, making them promising targets for immunotherapy. Additionally, employing strategies to reduce ITH, such as combination therapies that target both CSCs and cancer cells, may improve the effectiveness of immunotherapeutic interventions.

## 4. Inherent Strategies of Immune Evasion by CSCs

**Figure 3:**
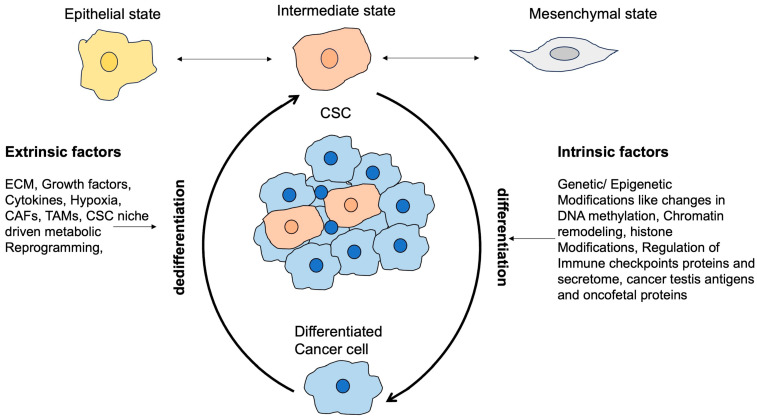
Intrinsic and extrinsic factors that influence the plasticity of CSCs.

Cancer stem cells (CSCs) have developed various mechanisms to evade detection and destruction by the immune system. One strategy they employ is the upregulation of programmed cell death ligand 1 (PD-L1), which contributes to an immunosuppressive tumor microenvironment (TME) by suppressing the activation of T cells [99]. Additionally, CSCs downregulate MHC-I molecules, allowing them to evade recognition by cytotoxic T lymphocytes (CTLs). This dual approach helps CSCs evade immune response and contributes to tumor progression [100]. Another strategy is to alter their epigenetic changes, which enables CSCs to alter their gene expression by upregulating the genes that assist in immune evasion and induce apoptotic resistance [101]. CSCs alter their secretions of exosomes, cytokines, and chemokines. By altering their secretome, CSCs recruit MDSCs and Treg cells, which creates an immunosuppressive TME [102]. The overexpression of oncofetal proteins and cancer testis antigens (CTA) by CSCs helps in immune evasion. Through these various intrinsic mechanisms, CSCs evade immune detection (Figure 3). There is an urgent requirement to develop innovative and multifaceted therapeutic strategies to overcome these immune evasion strategies. Table 1 outlines various immune evasion and immune suppression strategies of CSCs observed in different cancers.

The dynamism of CSC plasticity is a pivotal aspect of intratumor heterogeneity, allowing cells to transition between CSC and differentiated or non-CSC states reversibly. Genetic or epigenetic changes and environmental factors, including injury, inflammation, and senescence, primarily drive this plasticity. CSCs are notable for activating EMT, often exhibiting an intermediate state during this transition. The underlying mechanisms of these transitions are influenced by genetic and epigenetic modifications regulated by the TME, including cytokines and interactions with CAFs and TAMs. A complex interplay of intrinsic and extrinsic factors modulates CSC plasticity. On the intrinsic side, genetic mutations in key transcription factors and genes of signaling pathways combine with epigenetic changes including chromatin remodeling, DNA methylation, and the involvement of non-coding RNAs, to influence cellular behavior. Extrinsically, the TME is critical, as it encompasses a diverse cellular landscape that includes CSCs, differentiated cancer cells, immune cells, and various stromal components, along with non-cellular elements, like the ECM, regions of hypoxia, nutrient deprivation, cytokines, growth factors, and metabolites. Together, these intrinsic and extrinsic signals intricately regulate CSC plasticity, stemness, and metabolic adaptations, aiding in the immune evasion of CSCs.

### 4.1. Immune Checkpoint Proteins

Immune checkpoint proteins create an immunosuppressive TME by interacting with ligands of immune cells and inhibiting their activity. The inactivation of T cells by PD1–PDL1 interaction leads to immune evasion [113]. In hepatocellular carcinoma, MYC binds to PD-L1 and increases its expression, promoting an immunosuppressive TME, and inactivating MYC reduces PD-L1 expression, which enhances the antitumor response [114]. PD-L1, by regulating the β-catenin pathway, increases tumor stemness and cancer progression [115]. Aldehyde dehydrogenase 1A1 (ALDH 1A1) CSC marker expression is correlated with PD-L1 in breast cancer [116]. Increased PD-L1 expression in CSCs has also been reported in head and neck cancer [59], breast cancer [117], and colon cancer [118]. Another immune checkpoint protein, B7-H4, is overexpressed in glioblastoma stem cells [119]. CD276 and B7x are overexpressed in squamous cell carcinoma [65] and breast cancer [120] CSCs, which helps in immune evasion during cancer progression. Head and neck CSC CD 80 is expressed in the presence of TGF-β, which decreases cytotoxic T-cell activity [66]. CSC marker CD 24 binds to TAMs and promotes immune evasion [121]. The overexpression of CD47 on leukemia cells (LSCs) and hematopoietic stem cells (HSCs) assists in evading phagocytosis [122]. Dual targeting of PD-L1 and CD 47 immune checkpoints enhances antitumor immunity [123]. CSCs employ various strategies to escape the body’s immune response, facilitating tumor development and contributing to resistance against immunotherapies. Gaining insight into how CSCs evade immune detection is essential for creating more effective treatment options.

### 4.2. Major Histocompatibility Class (MHC) Molecules

The immune response is generated when cytotoxic T cells recognize the antigens presented by MHC-I molecules. Antigen processing and presentation is a complex process that involves proteasomal degradation of antigens, which are then transported to the endoplasmic reticulum and loaded on MHC-I molecules for antigen presentation on the cell surface to T cells. This process is crucial for generating an immune response [124,125]. CSCs regulate this antigen production and presentation by MHC-I molecules by downregulating the expression of transporter proteins and MHC-I molecules, like stem cells, to evade immune detection [68]. Studies have shown that in neuroblastoma and lung cancer, MHC-I expression is downregulated by polycomb repressive complex (PRC-2) through epigenetic mechanisms [126]. TGF-β signaling in CSCs suppresses antigen presentation, which was reported in SCC, promoting immune evasion [66]. CSCs in tumorspheres cultured from twelve different cancer cell lines showed reduced expression of HLA-I and II or lost their expression completely, coupled with lack of stimulation to interferon-γ (IFN-γ), leading to impaired antigen presentation. This process is critical for the immune evasion observed in CSCs [127]. Decreased expression of MHC-I molecules has also beenshown in CSCs of glioblastoma, melanoma, HCC, and lung cancer [31,111,128,129]. In head and neck cancer stem cells, many regulatory proteins, like TAP 2, MHC-I, HLA-II, and HLA-A2, involved in antigen processing and presentation were downregulated [104,130]. CSCs in melanoma decrease the expression of tumor-associated antigens along with MHC molecules [31,131]. In conclusion, CSCs employ different strategies to escape immune detection by modulating the expression of antigen processing and presentation regulatory proteins, which dampen T-cell recognition and foster a suppressive environment that supports CSC growth and tumor development. Gaining insights into how CSCs evade immune responses is essential for developing novel therapeutic strategies to counter these evasion tactics. 

### 4.3. Secretome Regulation by CSCs

The CSC secretome plays a pivotal role in drug resistance, invasive growth, and immune evasion [132]. Under physiological conditions, IL-33 is localized within the CSC nucleus, but in the presence of TGF-β, IL-33 is secreted into the extracellular environment via an NRF2-regulated mechanism [133]. This extracellular IL-33 is essential for directing bone marrow cell differentiation into macrophages. These macrophages, in turn, contribute to creating a suppressive environment that supports tumor development. Tumor suppressor protein P53 inactivation in the CSCs of liver cancer is associated with an abnormal increase in IL-34 secretion, which promotes the polarization of M2 macrophages via CD36 [134]. CSCs in cholangiocarcinoma secrete osteoactivin, IL-34, and IL-13, which aid in macrophage differentiation, leading to immunosuppression and tumor promotion [135]. CSCs in ovarian cancer were shown to augment the secretion of WNT and IL-10, which promote the M2 macrophage activation, thus inducing immune evasion [136]. In glioma, CSCs increased phosphorylation of STAT3-induced immunosuppression and evasion by secretion of MIC-1, TGF-β, CSF-1, IL-6, and IL-10 [119,137]. CSCs in colorectal cancer utilize IL-4 to shield themselves from chemotherapy-induced cell death while promoting the formation of M2 macrophages [138,139]. CSC-secreted CSF increases the formation of M2 macrophages, leading to tumor progression and immune evasion [140]. CSCs modulate the immune microenvironment by secretion of VEGF, which induces blood vessel formation and promotes resistance against immunotherapy [57,141]. CSCs in glioblastoma secrete histamine, which enhances angiogenesis in endothelial cells [142]. Hyperactivation of Wnt signaling drives the movement of lymphoma toward endothelial cells and promotes angiogenesis [143]. CSCs create an immunosuppressive microenvironment by secreting chemokine CXCL2, which recruits pro-tumorigenic neutrophils [144]. In breast cancer, CSCs secrete autocrine chemokine CXCL1, which activates pathways associated with immune evasion and tumor promotion by modulating the factors regulating tumor growth and immunosuppression [145]. Chemokines CCL1, CCL2, and CCL5 secreted by CSCs recruit tumor suppressive Treg cells and MDSCs [53,146,147]. The infiltration of T cells is inhibited by Wnt pathway activation and secreted chemokines CCL4 and CCL5, facilitating tumor growth in melanoma, lung, and colon cancer [148,149]. CSCs secrete autocrine chemokines CCL2, CSF2, IL6, and IL8, which activate the Wnt and NF-kB pathways to enrich CSCs and drug-resistant malignant cells [150]. Exosomes loaded with STAT3 signaling pathway proteins secreted by glioblastoma stem cells can induce the differentiation of monocytes to M2 macrophages by activating PD-L1 expression [151]. Metastatic melanoma-derived exosomes with PD-L1 induce the suppression of CD8^+^ T cells and promote tumor growth [152]. In conclusion, CSCs secrete chemokines, cytokines, and exosomes, which alter the TME, inducing immunosuppression and promoting tumor growth and immune evasion.

### 4.4. Epigenetic Alterations

Alterations in the epigenetic landscape of cancer play a vital role in the early stages of tumor development and the advancement and survival of both cancer cells and cancer stem cells (CSCs). These changes influence how tumors grow and adapt to their environment. Epigenetic reprogramming comprises changes in chromatin architecture, histone modifications, and DNA methylation. These alterations are crucial in CSC plasticity and immune evasion [153,154]. DNA methylase DNMT1 is reported to regulate cancer stemness in liver cancer and breast cancer [155,156,157]. Hypermethylation of TAP1 in breast cancer stem cells enhances immune evasion [158]. In LSCs, the inhibition of FTO demethylase reduced cancer stemness and increased antitumor immunity [159]. Histone demethylase LSD1 regulates cancer stemness and immune evasion [160,161]. LSD1 inhibition decreased CSC marker BMI1 and sensitized HNSCC to immunotherapy [162]. Chromatin remodeling complexes, such as the BAF complex (also known as the SWI/SNF complex), are known to regulate cancer stemness and immunity [163,164].

### 4.5. Oncofetal Proteins

Cancer cells can evade immune detection, a mechanism that shares a notable resemblance to immune tolerance mechanisms seen in testes and embryos. CSCs often exploit traits associated with the embryo and testes to shield themselves from immune attack. Cancer testis antigens (CTAs) are known to regulate cellular processes like the differentiation of stem cells and tumor initiation. The CTA score derived from a set of genes has been found to correlate with the cancer stemness score, and it is negatively correlated with immune infiltration [165,166,167,168]. In CSCs, several embryonic genes and developmental signaling pathways are reactivated, a phenomenon known as oncofetal drivers [169]. Transcription factors such as NANOG, OCT4, and SOX2 are overexpressed in several cancers, including liver, squamous, lung, brain, colon, and breast cancers [170,171,172,173,174,175]. These oncofetal proteins are reported to induce an immunosuppressive TME [176]. Cancer stem cells (CSCs) exploit these complex mechanisms to escape immune detection, thereby enhancing tumor growth and resistance to therapies.

## 5. Extrinsic Strategies of Immune Evasion by Cancer Stem Cells

Extrinsic factors are crucial in reshaping the CSC niche, which in turn fosters an environment conducive to immune evasion by secreting cytokines such as TGF-β, recruiting immune-suppressive cells like M2 macrophages and Treg cells, and creating a TME in which it is metabolically challenging for the immune system to combat cancer [42,177]. The CSC niche is a complex and dynamic microenvironment comprising cancer-associated fibroblasts (CAFS), pericytes, endothelial cells, stromal cells, immune cells, chemokines, cytokines, and metabolites [178]. Non-CSCs can be manipulated by CSCs to aid in tumor growth.

### 5.1. Immune Cells

**Figure 4:**
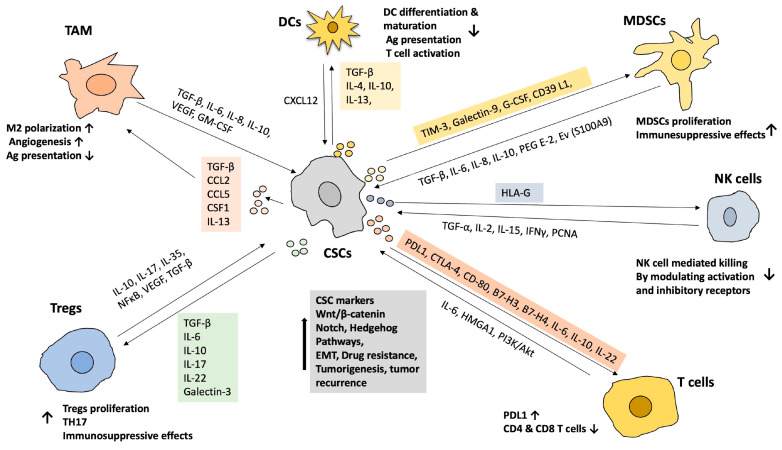
Crosstalk between CSCs and immune cells in the TME.

CSCs and immune cell interactions are crucial in inducing tumor progression and immune evasion in the CSC niche. Immune suppressive cells like Tregs, MDSCs, and M2 macrophages regulate cancer stemness, which increases therapeutic resistance (Figure 4). In liver cancer, M2 macrophages maintain cancer stemness and induce exhaustion of CD8^+^ T cells by secreting TGF-β [179]. In squamous cell carcinoma (SCC), TGF-β secreted by macrophages induces aggressiveness and drug resistance [133]. MDSCs induce cancer stemness in ovarian cancer by increasing microRNA-101 [180]. MDSCs increase CSC properties by activation of STAT3 and NOTCH signaling in breast cancer [181]. In breast cancer, T cell-secreted cytokine IFN-γ induced cancer stemness by converting non-CSCs to CSCs [182]. In NSCLC, low-dose IFN-γ induced cancer stemness [183]. IL-17 secreted by T cells induces cancer stemness in gastric, pancreatic, and ovarian cancers [184,185,186]. T cells can induce CSC properties by interacting with breast cancer cells [187]. CAFs-induced cancer stemness in bladder cancer [188]. In breast and lung cancer, subsets of CAFs promoted cancer stemness and chemoresistance by secreting IL6 and IL-8 [189].

CSCs play a significant role in modulating immune functions, influencing the immune response either directly or through the secretion of cytokines. CSCs undermine the activity of antitumor T cells by utilizing immune checkpoint molecules such as MHC-I, PD-L1, and CD80. They also hinder the maturation and differentiation of DCs through the mechanisms involving TGF-β and the expression of HLA-G. Interestingly, NKG2D ligands can selectively target and eliminate CSCs that lack MHC-I expression, operating independently of APCs. However, inhibitory ligands on NK cells interact with CSCs, suppressing the activation of NK cells. Moreover, CSCs recruit and polarize specific T helper cell subsets, particularly TH17 and Treg cells, through the release of cytokines and chemokines. Treg cells, in turn, secrete TGF-β1and IL-17, enhancing their functions and promoting self-renewal in CSCs and expression of stem cell markers contributing to tumor progression. Additionally, CSC-derived PD-L1 facilitates the infiltration of Treg cells into the TME. CSCs polarize M1 macrophages to M2 macrophages. The presence of macrophages and MDSCs in the TME further regulates T cells. Their immunosuppressive effects are partially mediated by factors secreted by CSCs, such as TGF-β1, PEG-E2, etc. Furthermore, CSCs can express TIM-3 and Galectin-9, promoting the expression of MDSCs. Together, these intricate interactions reshape the TME, creating a niche that supports the growth of CSCs.

### 5.2. Mechanotransduction Signaling

In the CSC niche, mechanotransduction signaling is vital in enabling immune evasion in the TME. Mechanical properties of ECM, including elasticity and stiffness, greatly influence differentiation, proliferation, invasion, and migration via mechanotransduction. In HCC, DDR1 signaling induced cancer stemness by inhibiting Hippo signaling [190]. DDR1 inhibited the infiltration of immune cells in breast cancer [191]. Increased ECM stiffness inhibited T-cell activation and decreased cytokine secretion via YAP signaling [192]. Even soft ECM was shown to inhibit T-cell cytotoxicity, enabling tumor immune evasion [193]. In the TME, integrin signaling regulates cancer stemness and induces resistance to immunotherapies [194]. In breast cancer, integrin signaling maintained cancer stemness and induced chemoresistance [195]. In conclusion, the biomechanics of the CSC niche regulates cancer stemness and immune cell function.

### 5.3. Metabolic Reprogramming

Under aerobic conditions, tumors enhance the glycolytic pathway, known as the Warburg effect, to maintain their malignancy and tumor growth [196]. In contrast, cancer stem cells (CSCs) primarily depend on oxidative phosphorylation (OXPHOS) for energy needs. This metabolic pathway provides various benefits, such as resilience against inhibitors that target glycolysis and employ fatty acid oxidation (FAO) to support their survival and metabolic adaptability [197,198,199]. As a result, CSCs utilizing OXPHOS may possess a selective edge in TME since they can use scarce nutrients more efficiently. Lactate produced by non-CSCs via glycolysis can be a beneficial energy source for OXPHOS in CSCs, fostering metabolic symbiosis [200]. This metabolic symbiosis creates a supportive TME and increases the cancer stemness and survival of CSCs, promoting tumor growth. However, like cancer cells, CSCs’ metabolic traits are not static. In CSCs, both glycolysis and OXPHOS can coexist. The state of the cells and the external factors present in the TME shape these metabolic characteristics [201,202,203,204,205]. For example, glycolysis is essential in CSCs’ quiescent state and aids in self-renewal, maintaining stemness, and antioxidative capabilities [206,207]. Furthermore, tumors exhibit high glucose consumption via glycolysis, which can hinder T-cell functions and promote tumor growth [208,209]. Key regulators of metabolic pathways, such as mTOR, HIF-1α, and PPARγ, are known to regulate immunometabolism and have also been linked to Treg cell differentiation. This suggests a complex relationship between metabolic regulation, CSCs, and immune cells [210,211,212,213].

Treg cells, the key regulators of the immune response, may significantly impact the immune environment within the CSC niche, potentially aiding in tumor progression and immune evasion [214]. An intriguing illustration of metabolic reprogramming is observed in PDAC. When the primary energy source shifts from glucose to galactose, PDAC cells are driven to adapt their metabolism toward OXPHOS. This metabolic switch enhances cancer stemness, invasiveness, and immune evasion [215]. In breast cancer, leptin secreted by adipocytes regulates therapeutic resistance and self-renewal ability of CSCs by activation of JAK/STAT3 signaling pathway, which leads to increased levels of the FAO enzyme carnitine palmitoyl transferase 1B [216]. In cervical cancer, acetyl-CoA, a metabolite of FAO, was shown to increase the acetylation of histone H3 on the promoters of stemness genes OCT4, SOX2, and NANOG, thereby further enhancing the cancer stemness and lymph node metastasis [217]. In colon cancer, kynurenine produced by IDO1 and TDO2 enzymes was shown to hinder immune surveillance and promote cancer stemness and liver metastasis via the TDO2-kynurenine-AHR pathway [218]. Furthermore, in colon cancer, serotonin or 5-hydroxytryptamine, a neurotransmitter, promotes CSCs self-renewal by regulating Wnt signaling [219]. Studies have shown that serotonin also modulates tumor immunity [220]. These unique metabolic adaptations of CSCs, characterized by metabolic plasticity and interactions with metabolites in the CSC niche, are crucial in enhancing their survival, maintaining their cancer stem-like properties, and aiding in their ability to evade the immune system. Table 2 lists various drugs currently in clinical trials that target the CSC niche.

## 6. Overview of Cancer Treatment: Conventional Methods to Innovative Strategies

Cancer therapies have evolved dramatically over the past few years, including traditional methods such as surgery, chemotherapy (CT), and radiation therapy (RT). Conventional methods remain pivotal in combating cancer, but they often face challenges like tumor heterogeneity, intrinsic or acquired resistance, and potential toxic side effects. RT targets cancer cells either by inducing DNA damage or by ROS generation. CSCs have enhanced DNA repair capabilities and lower levels of ROS [221,222]. CSCs exhibit this radioresistance due to DNA damage response activation in glioma stem cells [223]. In breast cancer, CSCs show low ROS levels due to an antioxidant profile that promotes radioresistance. Radiation is typically administered in multiple fractions, which allows CSCs to re-enter the cell cycle, which can lead to tumor repopulation. While most CSCs are in the quiescent G0 phase and slowly proliferate, tumor repopulation is frequently implicated in treatment failure. This occurs when the tumor regrows after receiving the sublethal doses, outpacing the growth rate of the untreated tumor [224].

**Figure 5:**
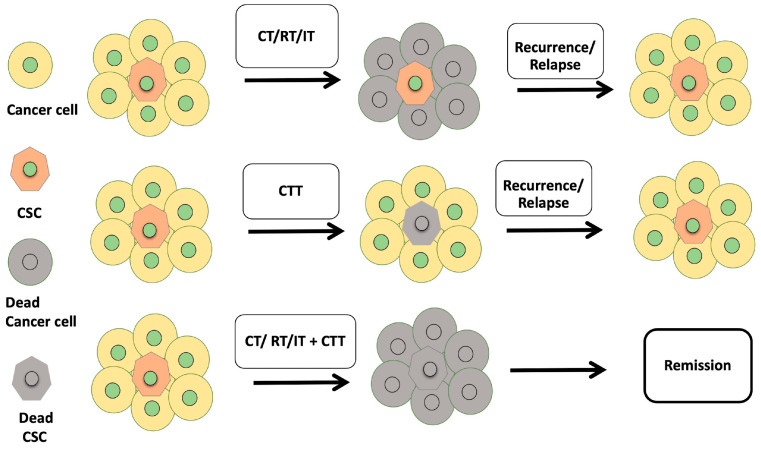
Targeting cancer stem cells (CSCs) in combination with innovative strategies.

CT and RT generally target fast-growing malignant cells; however, CSCs are dormant, proliferate slowly, and are not eliminated. Several chemotherapeutic drugs are known to induce cancer stemness. For example, FDA-approved drugs like cisplatin and tamoxifen are known to induce cancer stemness in HNSCC, NSCLC, ovarian cancer, and breast cancer [225,226,227,228,229,230,231]. Similarly, studies have reported that radiation induces cancer stemness and metastasis, as well as modulates the metabolism of cancer cells [232,233,234]. Enrichment of CSCs upon chemotherapy and radiation therapy leads to therapeutic resistance. Studies have shown that immunotherapy induces cancer stemness, and CSCs are known to evade immune detection [182,235]. Innovative approaches like combinatorial treatment of chemotherapy and radiation therapy with CSC-targeting compounds like diallyl trisulfide (DATS) [236], fisetin [237], and silibinin will enhance therapeutic efficacy [238]. Compounds like DATS and fisetin have been proven to target both proliferating cancer cells and quiescent CSCs [236,239,240,241]. Silibinin is a radiosensitizer that targets cancer cells and cancer stem cells [242,243,244]. Combining phytochemicals such as DATS, fisetin, and silibinin with immunotherapy could be an innovative approach to target CSCs and enhance treatment outcomes (Figure 5). Table 3 provides a list of different drugs that target CSCs across various types of cancers.

CSC enrichment following chemotherapy (CT), radiation therapy (RT), and immunotherapy (IT) often results in treatment resistance. To combat this challenge, innovative strategies that combine traditional therapies, including immunotherapy with CSC-targeting phytochemicals, mAbs, or any CSC marker-specific drugs or immunotherapies like DC vaccines, immune checkpoint inhibitors, CAR-T, CAR-NK, CAR-macrophage, NK, or oncolytic virotherapy, could improve and enhance treatment outcomes (CTT–CSC-targeted therapy).

**Table 3:** List of different drugs targeting CSCs in different cancer types.

S.No	Cancer	Target	Drug/compound	Mechanism
1	Breast cancer	Dopamine D2 receptor	Sulpiride (SUL)	Inhibits CSCs in vitro and in vivo [245]
2	Osteosarcoma	Wnt/β-catenin	IWR-1	Inhibits CSC self-renewal ability and expression of CSC markers [246]
3	Lung cancer	NOTCH3 signaling	Evodiamine (EVO)	Inhibits CSC proliferation [247]
4	Esophageal Adenocarcinoma	YAP1	CA3	Inhibition of CSC sphere formation and decreased ALDH1^+^ cells [248]
5	Breast cancer	Ferroptosis	Ironomycin (AM5)	Induces ferroptosis in bCSCs [249]
6	Breast cancer	Ferroptosis	Salinomycin	Induces ferroptosis in bCSCs [249]
7	Glioblastoma	Wnt, Notch, Hedgehog	Ajoene	Reduces cancer stemness in glioblastoma [250]
8	HNSCC	CD44, CD133, ALDH1, SOX2, OCT4	DATS	Inhibits CSC sphere formation, reduces CSC fraction, and decreases SOX2 and OCT4 [236]
9	Lung cancer	CD44, CD133	Fisetin	Downregulates CD44 and CD133 CSC markers [237]
10	Sarcoma	HDAC inhibitor	MC1742 and MC2625	Inhibits CSC proliferation [251]
11	HNSCC	cGAS-STING and BMI^+^ CSCs	PTC209/MnO2@BSA nanoparticles (PMB NP)	PMB NPs increase cGAS-STING, T cell-mediated immune response; reduce CSCs and EMT [252]
12	Breast and pancreatic cancer	CD44	Iron oxide magnetic nanoparticles + anti-CD44 antibody	Inhibition of CSCs growth [253]
13	Osteosarcoma	CD133	Salinomycin + nanoparticles + CD133 aptamers	Inhibits CD133^+^ CSC sphere formation and proliferation [254]
14	Breast & colon cancer	CD44	PLGA-c-PEG + Paclitaxel	Enhances efficacy against CSCs [255]
15	Colon cancer	ALDH	Paclitaxel + nanoparticles of cetyl alcohol	Decreases cancer stemness [256]

## 7. Innovative Therapeutic Approaches Targeting Cancer Stem Cells Through Immune-Based Modalities

Recent progress in cancer research and cell biology, particularly the molecular mechanisms governing immune responses, has paved the way for developing immunotherapies. These therapeutic strategies can be divided into active immunotherapy and passive or adaptive immunotherapy. Active immunotherapy involves genetically engineered macrophages, B cells, and dendritic cells to stimulate an immune attack against cancerous tumors. In passive immunotherapy, monoclonal antibodies (mAbs), genetically modified NK cells, and T-cells are used. Checkpoint inhibitors and mAbs have emerged as significant advancements in cancer treatment, particularly for patients with metastatic or advanced stages. Immunotherapy boosts the immune system’s capacity to identify and destroy cancer cells. However, when it comes to targeting CSCs with immunotherapy, distinctive challenges arise. CSCs can often evade immune detection through various mechanisms, including downregulating immune checkpoint proteins and MHC molecules, secretion of immunosuppressive factors, immune suppressive cells like Tregs and MDSCs, and metabolic and epigenetic reprogramming [257]. Given the pressing need for improved immunotherapeutic strategies, recent efforts have begun to focus on distinct characteristics of CSCs to develop more effective treatments [258]. In subsequent sections, we will review innovative immunotherapeutic approaches to target CSCs and their eradication.

### 7.1. Dendritic Cell Vaccines in Cancer Immunotherapy

A widely studied approach in adoptive immunotherapy focuses on the role of APCs in presenting antigens, which in turn helps activate T cells. Dendritic cells (DCs) are critical in initiating innate and adaptive immune responses among these APCs. DCs activate CD4^+^ T cells by presenting antigens through MHC-II. Additionally, they can engage CD8^+^ T cells through a process known as cross-presentation [259]. The transfer of DCs loaded with TAAs into patients activates T cells. This activation is crucial in stimulating the immune system, enabling it to effectively recognize and destroy cancer cells. This cancer vaccination approach positions CSCs as potential antigens to provoke immune responses [260,261]. Recent advances in targeting CSCs have categorized DC vaccines into four groups: inactivated CSC/CSC lysate-based vaccines, DC vaccines loaded with CSC lysate, DC vaccines loaded with CSC lysate generated cytotoxic T-cell vaccines, and experimental models for both prophylactic and therapeutic combinational strategies [262]. Studies have shown that CSC lysates can elicit robust T-cell responses, offering the advantage of targeting multiple antigens simultaneously [263]. Vaccination with CSC-based strategies prevented melanoma metastasis in the lungs and inhibited the progression of SCC [264]. DCs loaded with CSC lysates enhanced the survival of mice and led to tumor suppression [265]. Infusion of DCs loaded with CSC lysates stimulated the expansion of CD8^+^ T and CD45^+^ T cells in breast cancer tumor mouse models [266].

In studies using mouse models of SCC7 squamous cell carcinoma and D5 melanoma, vaccination following surgery with dendritic cells loaded with ALDH-high SCC7 cancer stem cells showed promising results. This approach reduced the likelihood of tumor recurrence and improved overall survival rates in the subjects. Specifically, in D5 melanoma mouse models, tumor growth and lung metastasis were inhibited and increased survival [267]. Furthermore, triple combination therapy, which includes the CSC-DC vaccine and PD1 and CTLA-4 blockade, has been shown to enhance the T-cell response against CSCs and reduce TGF-β secretion in melanoma mouse models [268]. Combining immunotherapy and chemotherapy has demonstrated promise in enhancing antitumor activity and overcoming therapeutic resistance. Combining the CSC-DC vaccine with cisplatin-induced apoptosis in the Ehrlich carcinoma mouse model reduced tumor growth and downregulated multidrug resistance (MDR) and Bcl2 gene expression [269]. CSC-loaded DCs effectively stimulated cytotoxic T cells against CSCs and improved survival rates of 9L CSC brain tumor mouse models [270]. In breast cancer, RNA is isolated from breast cancer cells and CSCs. Interestingly, DCs exposed to RNA isolated from CSCs demonstrated an enhanced ability to activate effector T cells and induce apoptosis in breast cancer cells. However, CSCs are resistant to apoptosis induced by T cells due to the upregulation of PD1 on CSCs [200]. The preclinical investigations of DC vaccines show promise, providing a deeper understanding of various DC subtypes, strategies to overcome an immunosuppressive TME, and the identification of new biomarkers to enhance the efficacy of these vaccines.

### 7.2. Chimeric Antigen Receptor (CAR) Based Immunotherapy

#### 7.2.1. CAR-T Cell Therapy

Genetically engineered cells have shown significant promise in targeting CSCs with various immunotherapy approaches. T cells, which are vital for mediating the immune response against tumors, can be modified to enhance their efficacy. Among these modifications, CAR-T cells have yielded encouraging clinical results, particularly in combating hematologic malignancies. CAR-T cells are designed to recognize and attack cells expressing the CD19 antigen on specific leukemia cells [271]. The process involves collecting T cells from either patients or healthy donors, which are then modified ex vivo to express CARs that target tumor-associated antigens (TAA). Once suitably engineered, these are injected into the patient, allowing for a tailored immune response against cancer cells. CAR-T cells can recognize target cells regardless of the expression of MHC molecules. Targeting CSCs with CAR-T cell therapy may thus enhance cancer treatment outcomes [272]. Preclinical research has yielded promising outcomes in utilizing CAR-T cells to combat CSCs. For instance, CAR-T cell therapy has been evaluated in glioblastoma as a standalone treatment and combined with standard chemotherapy regimens in gastric and ovarian CSCs [273,274,275]. Clinical investigations have assessed the effectiveness of CAR-T cells directed against specific targets in various cancers, including AML, ALL, ovarian, colorectal, pancreatic, liver, brain, and breast cancers [276,277]. In a clinical trial involving patients with diverse malignancies, including HCC, CRC, and pancreatic cancer, CAR-T cells targeting CD133^+^ CSCs effectively eliminated them while maintaining manageable toxicity [277]. Fourth-generation anti-CD133-CAR4 T cells targeting CD133^+^ cholangiocarcinoma (CAA) cells demonstrated dose-dependent efficacy in their elimination [278]. CAR-T cells targeting EpCAM, which is upregulated in ovarian cancer, CRC, and peritoneal carcinomatosis, have shown remarkable success in eliminating EpCAM-expressing cancer cells [279,280]. Administration of EpCAM-CAR-T cells in CRC xenograft inhibited tumor growth and increased cytotoxic cytokines tumor necrosis factor-α (TNF-α) and IFN-γ [281]. Recent advancements have introduced the concept of multitarget CAR-T cells. Trivalent CAR-T cells engineered to recognize HER2, IL13Rα2, and EphA2 in glioblastoma samples can effectively address the antigenic heterogeneity and improve therapeutic outcomes in xenograft models [282]. However, it is essential to acknowledge that despite the promising nature of CAR-engineered immune cells targeting specific antigens, severe toxicities have been reported following their administration. The challenge of on-target or off-tumor toxicity may be exacerbated when targeting multiple antigens, given that many are also present on normal cells [283,284].

Advancing methodologies that can accurately identify tumor-specific antigens could significantly enhance the efficacy of CAR immunotherapies. Exploring the development of other engineered T-cell types, such as T-cell receptor (TCR) engineered cells, TCR-like CARs, and TCR-CARs aimed at targeting CSCs, could prove fruitful [285]. CSCs typically harbor mutations that result in neoepitopes displayed on their surface. This highlights a potential avenue to address the limitations of CAR design, as TCR-based CARs may uniquely target CSC-specific neoepitopes, potentially reducing the off-tumor toxicities. Other strategies to enhance safety by mitigating off-tumor toxicity may include refining CAR affinity. By optimizing the binding strengths of CARs, high-density TAAs could be effectively recognized, while low-density TAAs on normal cells would be ignored. Another potential approach involves using antigen-specific inhibitory CAR-T cells, which would express inhibitory receptors targeting normal antigens alongside TAA-specific receptors. This strategy could enable inhibitory signals to diminish cytotoxic responses when encountering normal cells that express TAAs. CAR-T cells that express an anti-CAR construct could be an additional strategy for controlling toxicity. Despite these innovative strategies, more comprehensive research is needed to better characterize these underexplored approaches to reducing toxicity in CAR-T cell therapies targeting CSCs.

#### 7.2.2. CAR-Macrophage (M) Cell Therapy

Limited filtration of effector immune cells in the TME poses a significant challenge to the effectiveness of immunotherapy. In innate immunity, monocyte-derived macrophages can penetrate tumor tissues. Recent advancements in our understanding of the TME have led to innovative strategies that utilize modified macrophages to counteract an immunosuppressive TME. Genetically engineered macrophages (GEMs) can be modified to secrete TGF-βR2 to diminish immunosuppression or to produce IL-21 to activate immune cells [286]. Recent preclinical research has shown encouraging results regarding the effectiveness of immunotherapies that utilize macrophages in suppressing tumors. Despite these promising findings, considerable work remains to enhance the efficacy and safety of CAR-M therapies before they can be applied in clinical settings [287]. CAR-Ms are engineered to have sustained proinflammatory M1 phenotype and show enhanced expression of proinflammatory cytokines and chemokines, improving their ability to present antigens and resist immunosuppressive signals from the TME. CAR-Ms demonstrated a notable ability to reduce the tumor burden and extend overall survival [288]. Utilizing edited macrophages for immunotherapy, especially against solid tumors, shows great promise for future investigations. Nevertheless, research on CAR-Ms targeting CSCs is currently lacking. Exploring this avenue could be a valuable direction for upcoming studies.

#### 7.2.3. CAR-NK Cell Therapy

In innate immunity, NK cells are crucial players, offering unique benefits over CAR-T cells. One notable advantage of CAR-NK cells is their reduced likelihood of provoking graft-versus-host disease, alongside a shorter lifespan compared to T cells, which helps limit unintended toxic effects on healthy tissues. Currently, NK-92 cell therapies are undergoing clinical trials to treat patients with AML, and early case reports indicate that these therapies are generally well-tolerated [289]. Third-generation CAR-NK 92 cells targeting CD133 in ovarian cancer have significantly inhibited tumor progression [290]. Notably, combining these CAR therapies with cisplatin has enhanced cell-killing efficacy more than using either treatment alone [290]. Scientists have developed CAR-NK cells that can simultaneously target CD24 and mesothelin. This dual targeting makes them particularly effective against ovarian cancer stem cells and non-stem tumor cells [291]. The combination of CAR-NK-92 and regorafenib has enhanced the anticancer effects in CRC mouse xenografts compared to monotherapy [292]. In multiple myeloma (MM), CAR-NK cell administration induced apoptosis in CSCs. CAR-NK targeting CS1 in combination with daratumumab (anti-CD38) in MM was shown to have promising anticancer effects and inhibit MM relapse by eliminating MM CSCs [293].

### 7.3. NK Cell Therapy

NK cells have the intrinsic ability to induce cytotoxic effects on damaged and cancer cells. Due to their diverse characteristics and interactions with the adaptive immune system, B cells, and T cells, NK cells are vital in defending the body against different malignancies. Studies have shown the cytotoxic potential of NK cells in treating hematological malignancies, and both autologous and allogenic NK cells were shown to target solid tumors effectively. Studies reported that NK cell therapy can target and eliminate CSCs [294]. NK cells activated with IL-2 and IL-5 could recognize and destroy CSCs in glioblastoma, colon cancer, melanoma, and breast cancer [69,295,296]. Allogenic NK cells were shown to target and eliminate colorectal CSCs. Non-CSCs or differentiated tumor cells demonstrated reduced sensitivity to NK cells, a phenomenon tied to their lower expression of NKp30 and NKp44 ligands for NCR NK cell-activating receptors compared to CSCs [29]. The systemic administration of NK cells treated with IL-2 and HSP-70 crossed the blood-brain barrier and targeted glioblastoma cells in an induced glioblastoma multiforme (GBM) rat model [297]. Moreover, emerging evidence suggests that combining mAbs with NK cell therapy targeting CSC markers can significantly enhance cancer treatment outcomes and eliminate CSCs. Cetuximab incubated with pancreatic cancer cells improves the efficacy against CSCs through the antibody-dependent cell-mediated cytotoxicity (ADCC) ability of NK cells [298]. In colorectal cancer, bispecific single-chain fragment variable (scFv) killer engagers (BiKEs) against CD133, which recognize CD133 on CSCs and CD16 on NK cells, were shown to enhance NK cell therapy [299]. Table 4 provides an overview of different pharmacological agents that are currently being investigated in clinical trials for their potential to target surface markers associated with CSCs.

### 7.4. Monoclonal Antibodies (mAbs)

The FDA has approved various mAbs, like cetuximab (anti-EGFR) for epithelial cancer, rituximab (anti-CD20) for lymphoma, trastuzumab (anti-HER2) for HER2-positive breast cancer, and daratumumab (anti-CD38) for multiple myeloma, etc. These mAbs leverage the body’s immune response to eliminate targeted cancer cells through different mechanisms, including the activation of immune effector cells, antibody-dependent cell-mediated cytotoxicity (ADCC), apoptosis induction, complement-dependent cytotoxicity (CDC), and receptor-mediated signaling blockade [300,301]. Recent advancements in the study of mAbs have opened new avenues for targeting CSCs [302]. Anti-CD271 mAb targets a CSC biomarker in hypopharyngeal cancer CD271-positive cancer, and CSC cells in xenograft models reduce tumor growth via ADCC [303]. The Notch signaling pathway has also been found to protect chemoresistant CSCs. A phase IB clinical trial explored the viability of combining standard chemotherapy with demcizumab (anti-DLL4) to enhance antitumor efficacy [304]. In CSCs, ROR1, an oncoembryonic orphan receptor for Wnt5a, is overexpressed, particularly in neoplastic B cells of patients with CLL. The administration of cirmtuzumab (anti-ROR1) was shown to block gene expression signatures associated with stemness and effectively inhibit ROR1 signaling in patients with CLL [305]. CSCs overexpress the drug efflux protein ABCG2, which contributes to chemoresistance. In multiple myeloma, anti-ABCG2 mAb conjugated with epirubicin induced apoptosis with decreased Bcl2, PCNA, and CD31 and increased caspase-3 and Bax expression [306]. CSCs aberrantly expressed chemokine receptor CXCR4, which is correlated with drug resistance, angiogenesis, and tumorigenesis. In AML, anti-CXCR4 mAb is combined with radioimmunotherapy targeting CSCs in tumor xenografts, demonstrating promising results [307]. Table 5 lists various functionalized mAbs that target distinct antigens on CSCs.

### 7.5. Immune Checkpoint Inhibitors (ICIs)

CSCs tend to overexpress immunosuppressive molecules or immune checkpoints (ICs), such as PD-L1 and CTLA-4 [324,325]. ICIs function by enhancing the immune system’s activity and counteracting the overexpression of ICs in TME of cancer cells [326,327]. PD1, PD-L1, and CTLA-4 are among the most recognized ICs that can downregulate the immune response and have been the focus of various clinical studies. Through activating ICs, CSCs avoid detection by the immune system, and inhibiting ICs may foster a more robust immune response against CSCs [47]. CTLA-4 regulates T-cell activation. Ipilimumab blocks CTLA-4 activation. Clinical investigations have shown that combining chemotherapy with ipilimumab may enhance therapeutic outcomes in patients with lung cancer [326]. PD-1 is found on B cells and T cells. PD1 hampers the immune function when it binds to PD-L1 across different cancer types. ICIs like pembrolizumab, cemiplimab, and nivolumab specifically target PD-1 and PD-L1 interactions, promoting T-cell cytotoxicity toward cancer cells [328]. Furthermore, PD-L1 induces T-cell anergy, diminishing immune responses in different cancers. It promotes CSC properties through its interactions with HMGA1 and activation of MAPK and PI3K/AKT signaling pathways [329]. In breast cancer, the presence of PD-L1 is linked to elevated levels of cancer stem cell (CSC) markers, including BMI1, Nanog, and OCT4. This relationship is mediated through the PI3K/AKT signaling pathway [330]. PD-L1 expression correlates with enhanced proliferation and chemotherapy resistance in gastric cancer stem cells [331]. The simultaneous inhibition of the ICs PD-L1 and CTLA-4, combined with the administration of the CSC-DC vaccine, improved the anticancer efficacy in a mouse model of melanoma [268]. These emerging strategies, which focus on immune checkpoints in CSCs and associated signaling pathways that regulate their expression, could provide valuable insights and opportunities for advancing therapeutic effectiveness in cancer treatment. Table 6 lists various immunotherapy trials involving cancer stem cell targets.

### 7.6. Oncolytic Virotherapy

The use of oncolytic viruses represents a groundbreaking strategy in cancer immunotherapy, primarily because of their distinctive capability to replicate specifically inside cancer cells, ultimately leading to the destruction of these malignant cells. This approach not only targets the tumors directly but also enhances the body’s immune response to improve cancer treatment. Traditional, unmodified oncolytic viruses risk affecting both normal and tumor cells. However, advancements in genetic engineering have enabled the development of oncolytic viruses that specifically target cancer cells while sparing healthy ones. This selective infection ultimately leads to the elimination of cancer cells [332]. Tumor cells often exhibit compromised interferon pathways, rendering them more vulnerable to certain viral infections like myxoma and vesicular stomatitis viruses [333]. Studies have identified several viral families with inherent oncolytic properties, including Adenoviridae, Herpesviridae, Reoviridae, Poxviridae, Picornaviridae, Togaviridae, and Paramyxoviridae [333,334]. Among the approved oncolytic viruses, Talimogene Laherparepvec, commercially known as Imlygic, is a modified form of HSV-1 that has gained FDA approval for treating melanoma [334].

Another notable example is Oncorine, a genetically altered adenovirus used to treat head and neck cancer [335]. Oncolytic viruses are administered through intratumoral injection or systemically via the bloodstream. Mechanistically, they operate through both direct and indirect pathways. Directly, oncolytic viruses can infect tumor cells by recognizing specific biomarkers like CD64 and laminin that are upregulated on tumor cells and indirectly inducing an immune response via cytolytic cells to attack tumor cells [336]. Emerging data indicate that oncolytic viruses can potentially target CSCs in various cancers, including brain tumors [337]. In liver cancer, GP-73-regulated oncolytic adenovirus GD55 induced cytotoxic effects on liver cancer stem-like cells and inhibited tumor progression and angiogenesis in liver cancer xenograft models [338]. In brain tumors, the oncolytic adenovirus Delta-24-RGD targets the p16INK4a/Rb pathway in CSCs. Delta-24-RGD triggered autophagic cell death in brain tumor CSCs associated with the accumulation of LC3-II and Atg5 and improved overall survival in the glioma mouse model [339]. Engineered oncolytic reovirus effectively targeted and killed CSCs and non-CSCs in breast cancer tumor xenografts [340]. Zika virus (ZIKV) showed an oncolytic effect on CSCs in glioblastoma, particularly patient-derived GSCs in tumor organoids [341]. Studies have shown that ZIKV targets CSCs and stem-like cells in glioblastoma, ependymoma, and medulloblastoma by modulating the SOX2-integrin ανβ5 signaling [342]. While progress in the field is encouraging, it is essential to continue research efforts to address several key challenges. These include improving the methods for effectively delivering oncolytic viruses directly to tumors, ensuring precise targeting of CSCs, and boosting the stability and survival of these viruses in the bloodstream. These advancements are crucial for ensuring that therapeutic agents can reach tumor cells, even those in distant parts of the body. 

## 8. Challenges and Perspectives

Numerous challenges have been encountered in developing innovative therapeutic approaches to target CSCs. Addressing these challenges requires innovative delivery methods and strategies for enhanced precision in targeting CSCs selectively and minimizing toxicity toward normal healthy cells. Despite these efforts, finding unique CSC antigens or biomarkers remains a major hurdle. Current potential biomarkers can be categorized as intracellular and cell surface markers. Cell surface markers, especially signaling receptors and transport proteins, have gained attention for their role in diagnosing and delivering therapies to CSCs. However, these markers lack specificity, as they can also be expressed on healthy cells or non-CSCs. This overlap complicates their practical use in specifically targeting CSCs. On the other hand, intracellular markers, such as certain enzymes in CSCs, may offer promising targets [343,344,345]. For instance, ALDH is a key player that could be targeted with prodrugs activated exclusively in the presence of this enzyme, thereby selectively inhibiting CSCs [346]. The CSC transcription factors OCT3/4, SOX2, and BMI-1 are also interesting for designing targeted CSC therapies. However, it is essential to know that these transcription factors are not exclusive to CSCs, as many metabolic and signaling pathways are common to different cell types [347,348]. Additionally, while CSC-targeted therapies hold promise, significant challenges remain. For example, the expression of specific potential targets can vary due to factors like oxygen levels and cell density, complicating detection methods primarily reliant on immunohistochemistry and flow cytometry. The sensitivity of these targets to modifications further impacts detection accuracy. Conflict among CSC biomarkers raises valid concerns, as CSCs and their differentiated counterparts can initiate tumors [349]. Furthermore, CSCs exhibit remarkable plasticity, enabling them to adapt and survive even after targeted depletion. For instance, studies have shown that depleting LGR5+ CSCs may limit tumor growth, but tumors can still be maintained by LGR5 cells, which can revert to a CSC state when necessary [350]. To combat these challenges, combination therapy appears to be a viable strategy since it targets different pathways to address drug resistance and tumor heterogeneity. Although chemotherapies may not target CSCs directly, when used in conjunction with CSC-specific therapies, they could potentially reduce the risk of relapse. However, to maximize efficacy, careful consideration must be given to potential drug interactions and pharmacokinetics of combined treatments. While immunotherapy shows promise in attacking cancer cells, some CSCs may still evade the immune system. Some immunotherapy approaches rely on receptor and ligand interactions to target CSCs and spare specific heterogeneous subsets of CSCs that lack the corresponding ligand or receptor, allowing them to escape the effects of antigen-dependent therapies [351]. To tackle this, we need antibodies that can eliminate CSCs without necessarily identifying surface markers [352]. A significant obstacle in CAR-T cell therapy is the potential for on-target and off-target toxicity, which can harm healthy cells. In the case of NK cell therapy, challenges include the suboptimal performance of autologous NK cells and their limited persistence within the body over time [351,353]. To improve the outcomes, there is a growing interest in the adaptive delivery of NK cells to tumor sites, as these cells can potentially exhibit heightened efficacy. NK cell-derived extracellular vesicles show promise due to their ability to withstand the acidic pH in the TME, and their nanosized structure may offer favorable results in treating visceral tumors [354].

Additionally, nanoparticles loaded with anticancer drugs or imaging probes may facilitate targeted treatment and diagnosis of CSCs [355]. Salinomycin loaded onto chitosan-coated carbon nanotubes effectively targeted CSCs and inhibited self-renewal, invasion, and migration in gastric cancer [356]. This strategy aims to enhance the specificity of drugs for CSCs while minimizing the off-target effects. By increasing the drug payload and improving the ability of the drugs to penetrate biological barriers, nanotherapy can ensure more effective delivery. Furthermore, nanomaterials can carry multiple therapeutic agents, which can work together to create synergistic effects and potentially overcome drug resistance. The improved pharmacokinetic characteristics of these nanoparticles and their ability to shield drugs from enzymatic breakdown reinforce their role as a dynamic and effective tool in the fight against CSCs [357]. Integrating these novel therapeutic approaches with traditional methods could enhance overall cancer treatment efficacy, mainly through combination therapies that merge Immunotherapeutics with conventional treatments. Combining oncolytic viruses with chemotherapeutic agents may also be a promising avenue, as oncolytic viruses counteract the chemoresistance exhibited by CSCs, while chemotherapy potentially boosts the cytotoxic effects of oncolytic viruses [358]. To advance the development of effective therapies aimed at CSCs, in-depth studies into their unique properties and associated signaling pathways are essential. Utilizing high-throughput sequencing techniques and examining the expression profiles of CSCs could pave the way for innovative targeted treatment options.

## 9. Conclusions

CSCs are integral contributors to tumor biology; they can self-renew, evade immune detection, and exhibit resistance to therapies. These characteristics make CSCs critical in tumor growth, recurrence, and challenges associated with immunotherapy. Despite significant advancements in immunotherapies, the presence of the CSC niche is a key reason why complete tumor eradication remains elusive. The CSC niche actively regulates the interactions within the TME, fostering a dynamic setting that protects CSCs and alters the immune landscape to promote tumor survival. The heterogeneity and adaptability inherent in the CSC niche create an environment that can influence how these cells respond to immunotherapy. Recent concepts, like the resistance continuum observed in ovarian cancer treated with the PARP inhibitor Olaparib, highlight the gradual evolution of cell states facilitated by specific genetic and epigenetic changes. This framework suggests that when faced with immunotherapeutic pressure, CSCs may also experience progressive transitions supported by similar transcriptional and epigenetic reprogramming. Future research must determine if the transitions observed signify a distinct continuum of immune resistance in CSCs. Understanding this relationship could provide valuable insights into immune evasion mechanisms in these cells. The rapid emergence of advanced technologies such as 3D tumor models, lineage tracing, single-cell omics, spatial transcriptomics, and immune profiling enhances our understanding of CSCs [359]. These tools allow researchers to analyze CSC immune phenotypes and surrounding microenvironments in greater detail. For example, single-cell RNA sequencing can reveal the complex heterogeneity among CSCs and identify a subset of populations with unique immune evasion strategies. Spatial transcriptomics enables insights into how immune cells and CSCs are organized within the TME. Lineage tracing provides a means to observe CSC transitions in response to therapies, thereby shedding light on the developing resistance mechanisms over time. Furthermore, 3D tumor models and immunoprofiling are critical in mimicking the intricate nature of the TME. This method enables a more practical evaluation of therapeutic targets within environments resembling physiological conditions. By integrating these technologies, we enhance our comprehension of cancer stem cell (CSC) biology and pave the way for personalized treatment strategies. Utilizing high-resolution data allows researchers to discover therapeutic vulnerabilities and new CSC-specific biomarkers. Artificial intelligence and computational modeling can expedite the exploration of large datasets, revealing the underlying patterns contributing to therapy resistance and immune evasion. These findings are essential for developing combination therapies that target CSC plasticity while reprogramming the immune microenvironment to improve treatment effectiveness.

In conclusion, overcoming the therapeutic resistance associated with CSCs requires a comprehensive strategy that targets both their intrinsic plasticity and the dynamic interactions with the immune microenvironment. CSCs are specific subpopulations of cancer cells with self-renewal capabilities, and they play a vital role in tumor recurrence. Traditional treatments like chemotherapy and radiotherapy may effectively target the majority of tumor cells, but CSCs survive these interventions, leading to tumor relapse. This reality underscores the pressing need for novel therapeutic strategies. Current advances in single-cell spatial profiling and lineage tracing provide valuable tools to untangle the complexities of CSC behavior, enabling the rational design of combined treatment regimens. Such strategies aim to dismantle CSC survival niches while resensitizing tumors to immune attack, offering sustained remission in challenging malignancies. Immunotherapy modalities, including DC vaccines, NK cells, CAR-T cell therapies, mAbs, ICIs, and oncolytic viruses, may enhance the efficacy of existing cancer treatments by specifically targeting CSCs. However, these approaches come with challenges, emphasizing the need for more preclinical and clinical investigations to refine and advance these treatment paradigms. While these methods show promise, they also present several challenges, highlighting the necessity for further preclinical and clinical research. Such investigations are crucial for refining and enhancing these treatment strategies.

## Figures and Tables

**Table 1:** Overview of various immune evasion and immune suppression strategies used by CSCs in different cancer types.

S.No.	CSCs/Cancer Type	Molecular Target	Immune Cell	Mechanism
1	Pancreatic CSCs	CD44^+^ and CD133^+^	T cells	Pancreatic CSCs are linked to reduced infiltration of T cells and an elevated expression level of PD-L1, contributing to immune evasion [103]
2	HNSCC CSCs	CD44	T cells	HNSCC CSCs inhibit T-cell proliferation [104]
3	Lung CSCs	CD44^+^ and CD90^+^	T cells	Interactions between lung CSCs and T cells resulted in increased expression of CTLA-4, PD1, LAG3, and TIM-3 [105]
4	HCC CSCs	EpCAM	NK cells	EpCAM^+^ HCC CSCs are resistant to NK cell-mediated cytotoxicity [106]
5	Colon CSCs	Nanog	T cells	Colon CSCs are resistant to cytotoxic T lymphocyte-mediated killing [107]
6	Oral squamous cell carcinoma (OSCC) CSCs	CXCL12/CXCR4	Tumor-associated macrophages (TAMs), Cancer-associated fibroblasts (CAFs) and M2 macrophage-like monocytes	CAFs recruit monocytes via the CXCL12/CXCR4 pathway and induce differentiation into M2 macrophages, which promote the formation of CSCs in OSCC and enhance therapeutic resistance [108]
7	PDAC CSCs	CD90	Macrophages and monocytes	PDAC CSCs express increased PD-L1 and induce immunosuppression via monocytes and macrophages [109]
8	Glioblastoma CSCs	Macrophage migration inhibitory factor (MIF1) and arginase 1 (Arg1)	MDSCs and T cells	MIF1 released by CSCs induces Arg1 in MDSCs, which inhibits CTL response [110]
9	Melanoma CSCs	IL2	T cells and Treg cells	Melanoma CSCs inhibit IL-2-dependent T-cell activation and induce Tregs [31]
10	Glioblastoma CSCs	MHC molecules and NKG2D	T cells	Glioblastoma CSCs demonstrate lower immunogenicity and evade T-cell immune response [111]
11	Breast CSCs (bCSCs)	PD-L1	T cells	Increased PD-L1 expression on bCSCs helps in immune evasion [112]

**Table 2:** List of drugs currently in clinical trials targeting the CSC niche.

S.No.	Drug	Target	NCT Number
1	AZD-1480	JAK1/2	NCT01112397
2	Celecoxib	STAT3	NCT00087256
3	Tocilizumab	IL-6	NCT03999749
4	Pyrimethamine	STAT3	NCT01066663
5	Siltuximab	IL-6	NCT03315026
6	Reparixin	CXCR1	NCT01861054
7	Acalabrutinib	BTK	NCT04008706
8	Ibudilast (MN-166)	TLR4	NCT03782415
9	LCL-161	c-IAP	NCT01617668
10	Ipafricept (OMP-54F28	FZD receptor	NCT01608867
11	Vantictumab (OMP-18R5)	FZD receptor	NCT01957007
12	PRI-724	CBP/β-catenin	NCT01302405
13	WNT974	PORCN	NCT02649530
14	Fresolimumab	TGF-β1/2/3	NCT01472731
15	Galunisertib	TGF-βR1	NCT02688712
16	Lucanix	TGF-β2	NCT01058785
17	M7824	TGF-β/PD-L1	NCT04066491
18	Axitinib	VEGFR	NCT02853331
19	Bevacizumab	VEGFR	NCT02226289
20	AL101	γ-Secretase	NCT03691207
21	MK-0752	γ-Secretase	NCT00106145
22	Nirogacestat	γ-Secretase	NCT02109445
23	Demicizumab	DLL4	NCT02259582
24	Enoticumab	DLL4	NCT00871559
25	Entinostat	Arginase	NCT02453620
26	Decitabine	Arginase	NCT00030615
27	INCB001158	Arginase	NCT02903914
28	Ontak	CD25	NCT00726037
29	Zoledronate acid	Mevalonate pathway	NCT00588913
30	BMS-813160	CCR2/5	NCT04123379
31	Pexidartinib	CSF-1R	NCT02777710
32	AMG820	CSF-1R	NCT02713529
33	BL-8040	CXCR4	NCT02826486
34	ALX148	CD47/SIRPα	NCT03013218
35	IBI322	CD47/SIRPα	NCT04328831
36	Hu5F9-G4	CD47/SIRPα	NCT02216409

**Table 4:** List of various drugs targeting CSC-associated surface markers in ongoing clinical trials.

S.No.	Drug	Target	Condition	NCT Number
1	MGD006	CD123/CD3	Acute myeloid leukemia	NCT02152956
2	AMC303	CD44v6	Solid tumor	NCT03009214
3	XmAb14045	CD123/CD4	Hematologic malignancies	NCT02730312
4	Catumaxomabr	EpCAM/CD3	Ovarian cancer	NCT00189345
5	Tagraxofusp SL-401	CD123	Acute myeloid leukemia	NCT03113643
6	TTI-621	CD47	Solid tumor	NCT02663518
7	CSL362	CD124	Acute myeloid leukemia	NCT01632852
8	IBI188	CD47	Advanced malignancies	NCT03763149
9	CC-90002	CD47	Hematologic neoplasms	NCT02641002
10	AO-176	CD47	Solid tumor	NCT03834948
11	SRF231	CD47	Solid tumor	NCT03512340
12	Bivatuzumab mertansine	CD44v6	Metastatic breast cancer	NCT02254005
13	Vadastuximab talirine	CD33	Acute myelogenous leukemia	NCT01902329
14	RO5429083	CD44	Malignant solid tumors	NCT01358903
15	SPL-108	CD44	Ovarian cancer	NCT03078400

**Table 5:** List of various functionalized mAbs that target distinct antigens on CSCs.

S.No.	Monoclonal Antibody	Target	Cancer/CSCs
1	Cetuximab	EGFR	Pancreatic CSCS [298]
2	Demizumab	DLL4	Metastatic non-squamous cell lung carcinoma [304]
3	Trastuzumab	HER2	Breast CSCs [308]
4	Figitumumab	IGF	Colon CSCs [309]
5	Solitomab	EpCAM	Colon [310] and pancreatic CSCs [311]
6	Adecatumumab	EpCAM	Chemoresistant ovarian carcinoma [312]
7	AVE1642	IGF	Colon CSCs [313]
8	GV5	CD44	Human CSCs [314]
9	CSL362	CD123	Leukemic stem and progenitor cells (LSPCs) in chronic myeloid leukemia (CML) [315]
10	P245	CD44	Breast CSCs [316]
11	7G3	CD123	Acute myeloid leukemia stem cells [317]
12	OMP-52M51	Notch 1	Breast CSCs [318]
13	BH6H12	CD47	Brain tumor [319]
14	H4C4	CD44	Pancreatic CSCs [320]
15	Fusion of anti-CD3 scFv and anti-CD123 scFv	CD3 CD 123	Leukemia stem cells (LSCs) [321]
16	A1MCMMAF	5T4	CSCs in NSCLC [322]
17	H90	CD44	AML LSCs [323]

**Table 6:** List of various Immunotherapy trials involving cancer stem cell targets.

S.No.	Immunotherapy	Strategy	Condition	NCT number
1	T cell-based therapy	CD19 CAR-T	B-cell leukemia and lymphoma	NCT03398967
		CD123 CAR-T	CD122^+^ myeloid malignancies	NCT02937103
		CD22 CAR-T	Recurrent or refractory B-cell malignancy	NCT02794961
		CD22 CAR-T	B-ALL	NCT02650414
		CD33 CAR-T	Myeloid malignancies	NCT02958397
		CD33 CAR-T	CD32^+^ acute myeloid leukemia	NCT03126864
		CD38 CAR-T	B-ALL	NCT03754764
		CD138 CAR-T	Multiple myeloma	NCT03196414
		MUC1 CAR-T/PD-1 KO	Advanced esophageal cancer	NCT03706326
		EGFR IL-12 CAR-T	Metastatic colorectal cancer	NCT03542799
		MESO CAR-T	Refractory–relapsed ovarian cancer	NCT03916679
		MESO-19 CAR-T	Metastatic pancreatic cancer	NCT02465983
		LeY CAR-T	Myeloid malignancies	NCT02958384
		MOv19-BBz CAR-T	Recurrent high-grade serous ovarian cancer	NCT03585764
		LeY CAR-T	Advanced cancer	NCT03851146
		EpCAM CAR-T	Recurrent breast cancer	NCT02915445
		BCMA CAR-T	Multiple myeloma	NCT03767751
		IL13Rα2-CAR.T	Refractory malignant glioma	NCT02208362
		CD133-CAR.T	Liver cancer Pancreatic cancer Colorectal cancer Brain tumors Ovarian cancer Breast cancer	NCT02541370
		EGFRvIII-CAR.T	Malignant glioma Glioblastoma Gliosarcoma	NCT01454596
		EGFRvIII-CAR.T plus CD133-CAR.T	Cholangiocarcinoma	Case Report
		PSCA-CAR.T	Castration-resistant prostate carcinoma Metastatic prostate carcinoma, stage IV prostate cancer	NCT03873805
2	DC Vaccine	CSC-loaded DC vaccine	Colorectal cancer	NCT02176746
		mRNA from tumor stem cells with DC vaccine	Glioblastoma	NCT00846456
3	Checkpoint inhibitors	Atezolizumab + Bevacizumab, Sorafenib	Hepatocellular carcinoma	NCT03434379
		Anti-PD1/Anti-PDL1 mAb	Non-small cell lung carcinoma	NCT04977791

## Abbreviations

ACT	Adoptive cell therapies
APCs	Antigen-presenting cells
CAFs	Cancer-associated fibroblasts
CSCs	Cancer stem cells
CPT1B	Carnitine palmitoyl transferase 1B
CTLA-4	Cytotoxic T-lymphocyte antigen 4
CTLs	Cytotoxic T lymphocytes
DDR1	Discoidin domain receptor 1
DNMT1	DNA methyltransferase 1
ECM	Extracellular matrix
EVs	Extracellular vesicles
FTO	Fat mass and obesity-associated protein
FAO	Fatty acid oxidation
GBM	Glioblastoma
GSCs	Glioma stem cells
HCC	Hepatocellular carcinoma
HSV-1	Herpes simplex virus type-1
HLA-I and HLA-II	Human leukocyte antigen class I/II
ICB	Immune checkpoint blockade
IFN-γ	Interferon-gamma
IL-33	Interleukin-33
MHC	Major histocompatibility complex
MDSCs	Myeloid-derived suppressor cells
OXPHOS	Oxidative phosphorylation
PD-1	Programmed cell death protein-1
STAT3	Signal transducer and activator of transcription 3
TAAs	Tumor-associated antigens
TAP	Transporter associated with antigen processing
TAMs	Tumor-associated macrophages
TGF-β	Transforming growth factor beta
TME	Tumor microenvironment
Tregs	Regulatory T cells
VEGF	Vascular endothelial growth factor
